# Resonant Lasing Emission in Undoped and Mg-Doped Gallium Nitride Thin Films on Interfacial Periodic Patterned Sapphire Substrates

**DOI:** 10.3390/nano12183238

**Published:** 2022-09-18

**Authors:** Long Xu, Yuehan Cao, Tianwei Song, Caixia Xu

**Affiliations:** 1 Chongqing Key Laboratory of Micro & Nano Structure Optoelectronics, School of Physical Science and Technology, Southwest University, Chongqing 400715, China; 2 School of Primary Education, Chongqing Normal University, Chongqing 400700, China

**Keywords:** gallium nitride, lasing action, resonant structure, patterned sapphire substrate

## Abstract

In this work, low-threshold resonant lasing emission was investigated in undoped and Mg-doped GaN thin films on interfacial designed sapphire substrates. The scattering cross-section of the periodic resonant structure was evaluated by using the finite difference time domain (FDTD) method and was found to be beneficial for reducing the threshold and enhancing the resonant lasing emission within the periodic structures. Compared with undoped and Si-doped GaN thin films, p-type Mg-doped GaN thin films demonstrated a better lasing emission performance. The lasing energy level system and defect densities played vital roles in the lasing emission. This work is beneficial to the realization of multifunctional applications in optoelectronic devices.

## 1. Introduction

Gallium nitride (GaN) semiconductors have important applications in ultraviolet light sources, photodetectors, transistors, data storage, high-power electronic devices, and semiconductor lasers [1,2,3,4,5]. In terms of lasers, the advantages of GaN, such as reliable regulation and easy processing, have been developing rapidly [6,7]. For decades, blue light-emitting diodes were in short supply and urgently needed since *p*-type, wide-bandgap semiconductors could not be created in that state of technology. Excitingly, a highly *p*-type GaN layer was grown by S. Nakamura using the two-flow metal organic chemical vapor deposition (MOCVD) method, and high-brightness InGaN/AlGaN double-heterostructure blue light-emitting diodes were achieved [8]. After that, highly efficient room temperature blue, green, yellow, and white GaN light-emitting diodes with quantum well structures were designed [9,10]. Vertical-cavity surface-emitting lasers (VCSELs) have been exhibited in InGaN microcavities at a wavelength of 399 nm [11]. GaN-based vertical-cavity surface-emitting lasers have been widely used for optical storage, laser pointers, displays, and optical communications due to their low working current, high frequency operations, and easy fabrication [12,13,14,15,16]. Optically pumped stimulated emission from GaN thin films was studied at low temperature and room temperature [17,18], and ultraviolet blue lasing action was investigated in single GaN nanowires with axial Fabry–Perot modes (Q ≈ 103) [19]. The wurtzite structure of the GaN gives it very good thermal stability, and its non-centrosymmetric structure exhibits very good optical nonlinearities, which have great application prospects in optoelectronic devices [20,21]. At present, the growth and processing technologies of GaN mainly focus on molecular beam epitaxy, MOCVD, and laser etching technology [22,23]. In the material growth process, suitable buffer layers (such as AlN and AlInN) and an excellent annealing atmosphere are the keys to improving the film quality [24,25,26]. Although the roughness in high-quality GaN layers could be less than 0.1 nm, surface defects or bulk defects formed in metal-doped GaN films or thicker GaN films can lead to a decrease in film quality, which is still unavoidable in practical industrial applications. The carrier types, defects, and morphology play an extremely important role in the optical and lasing performance of the materials [27,28]. One limitation in the GaN material is that the high density of the defects degrades the efficiency and device performance in GaN-based LEDs, photodetectors, and lasers because of the lattice mismatch and heat accumulation between c-sapphire and the GaN active layer [29,30]. A series of recent studies indicated that artificially designed patterned sapphire substrate (PSS) has attracted great attention due to its significant advantages compared to the traditional flat sapphire substrate, which is good enough to compensate for losses by thermal and defect degradations [31]. Very recent studies found that a giant reduction in the threshold of the random lasing in organic perovskite thin films could be achieved on PSS since the multireflection, scattering, and resonance of light would occur within the cone structure of the PSS [32]. To further explore the optical enhancement of the GaN thin films in the lasing emission on the PSS, in this work undoped, Si-doped, and Mg-doped GaN thin films were grown on hexagonal lattice arrangement patterned c-plane sapphire (0001) substrates by using the MOCVD method. Sharp lasing emission was observed in the undoped and Mg-doped GaN thin films, and the physical picture based on the activation energy and defect levels was proposed.

## 2. Materials and Methods

**Figure 1:**
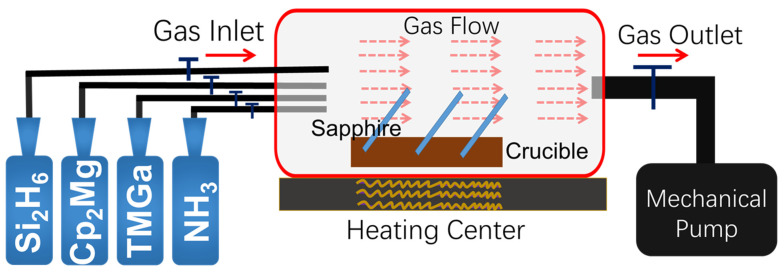
Schematic illustration of the growth of the undoped, Si-doped, and Mg-doped GaN thin films by using the MOCVD system.

Undoped, Si-doped, and Mg-doped GaN thin films were grown on hexagonal lattice arrangement patterned c-plane sapphire (0001) substrates by using the MOCVD method, as shown in Figure 1; trimethyl gallium (TMGa, Strem Chemicals Inc., Newburyport, MA, USA, 99.9999%), high purity ammonia (NH3, Rising Gas Inc., Chongqing, China, 99.9995%), bis(ethylcyclopentadienyl) magnesium (Cp2Mg, Aladdin, Los Angeles, CA, USA, 99.99%), and monosilane (SiH4, Lida Gas Inc., Chongqing, China, 99.999%) were used as the gas sources of gallium, nitrogen, magnesium, and silicone, respectively. Hexagonal lattice arrangement PSS was prepared with the dry etching technology, in which the photoresist coating, stepping, and UV exposure processes were first performed on the deep-cleaned flat sapphire substrate, and the PSS was then processed by using an inductively coupled plasma reactive ion etching (ICP-RIE) system in a hybrid Cl2/N2 gas atmosphere. Compared to the traditional wet etching method, this technology is close to being pollution-free and does not have excessive chemical residue. The prepared PSS substrates were inserted into the lots of the custom-built crucible and were put into the hot zone of the furnace. More details about the growth condition can be seen in Ref. [25] and Table 1.

The surface morphology of the undoped, Si-doped, and Mg-doped GaN thin films was investigated by using the scanning electron microscope (SEM, JEOL JSM-7100F, JEOL Ltd., Peabody, MA, USA). The room temperature ground state absorption spectra of the samples were measured by using a UV-Vis spectrophotometer (SHIMADZU Inc., Columbia, MD, USA, UV-2600). Additionally, the photoluminescence emission spectrum was excited by using a nanosecond laser centered at 266 nm (Changchun New Industry Inc., Changchun, China, DPS-266-Q/2~5mJ)) and recorded by a spectrometer (Ocean Optics Inc., Dunedin, FL, USA, QE65Pro). The carrier concentrations of the *p*-type Mg-doped GaN samples were measured by using a Hall measurement system (JouleYacht, HET-RT, Wuhan, China).

## 3. Results

**Figure 2:**
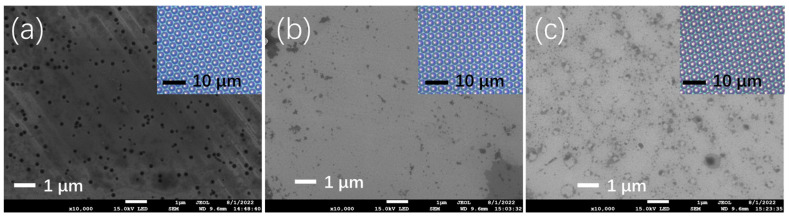
Scanning electron microscopy of the (**a**) Si-doped GaN, (**b**) undoped GaN, and (**c**) Mg-doped GaN thin film (the inset photographs were taken using a transmission optical microscope).

The surface morphology of the Si-doped, undoped, and Mg-doped GaN thin films was characterized by using a scanning electron microscope, as seen in Figure 2a–c, respectively. Numerous, large, three-dimensional hexagonal inverse pyramidal pits (V-pits) of approximately 100 nm could be observed randomly spreading in the Si-doped GaN thin films, as seen in Figure 2a and Figure S1 in the Supplementary Information. These V-pits might originate from the dislocation surface termination during the low-temperature growth [33] and act as energy barriers to affect the electronic and optical properties and efficiency of the LED devices [34]. The V-pits became much smaller (~20 nm) and less dense in the Mg-doped and undoped GaN thin films, as seen in Figure S2, which implied the decrease in the formation energies [35]. Very large, but low density, V-pits were found in the GaN thin film on the FSS, as seen in Figure S3, which confirmed that the PSS improved the quality of the GaN thin films and reduced the dislocation mismatch between the substrate and the active layers [36]. The inset photographs in Figure 2a–c were taken using a transmission optical microscope, showing the neatly arranged cones with a distance of 3.0 μm and a height of 1.5 μm.

**Figure 3:**
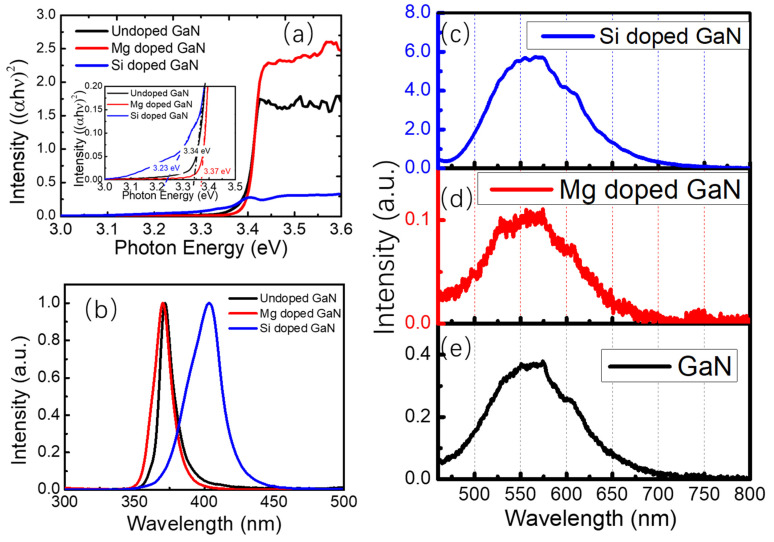
(**a**) Room temperature ground state absorption spectra of undoped, Mg-doped, and Si-doped GaN thin films (the inset is the bandgap calculated by using Tauc plot absorption curves). (**b**) Normalized photoluminescence of undoped, Mg-doped, and Si-doped GaN thin films excited by using a 355 nm continuous wave laser; defect luminescence spectra of (**c**) Si-doped GaN, (**d**) Mg-doped GaN, and (**e**) undoped GaN thin films.

Figure 3a shows the room temperature ground state absorption spectra of undoped, Si-doped, and Mg-doped GaN thin films. The bandgaps of the samples were estimated by using the intercept of the Tauc plot absorption curve with the x-axis. Compared with the bandgap of the undoped GaN thin films (3.34 eV), it increased to 3.37 eV in the Mg-doped GaN thin films and decreased to 3.23 eV in the Si-doped GaN thin films. This indicated that the magnesium ions and silicon ions were successfully doped into the GaN lattice structure. Normalized photoluminescence spectra of these samples were also recorded, as seen in Figure 3b; the emission peaks located at 370.3 nm (Mg-doped GaN), 372.3 nm (undoped GaN), and 403.6 nm (Si-doped GaN) were observed. The fluorescence emission centers of the undoped and Mg-doped GaN thin films were attributed to the radiative near-band-edge emission, and that of the Si-doped GaN came from the donor acceptor pair (DAP) recombination. To explore the energy structure in all the samples, the defect luminescence spectra of the samples were also studied at room temperature, as seen in Figure 3c–e. Broadband green luminescence (GL) and yellow luminescence (YL) could be seen in the defect luminescence spectra of the samples, corresponding to the Ga_N_ and N_Ga_ defects and the defect complex of V_Ga_-O_N_ [37,38,39]. The defect luminescence of the Si-doped GaN thin film was nearly 15 times more intense than that of the undoped GaN and 40 times more intense than that of the Mg-doped GaN, which implied different densities of defects in the thin films. All of these optical differences, such as bandgap, photoluminescence, and defect density, affected the luminescence and laser properties of the samples, and this will be discussed in more detail in the discussion section.

**Figure 4:**
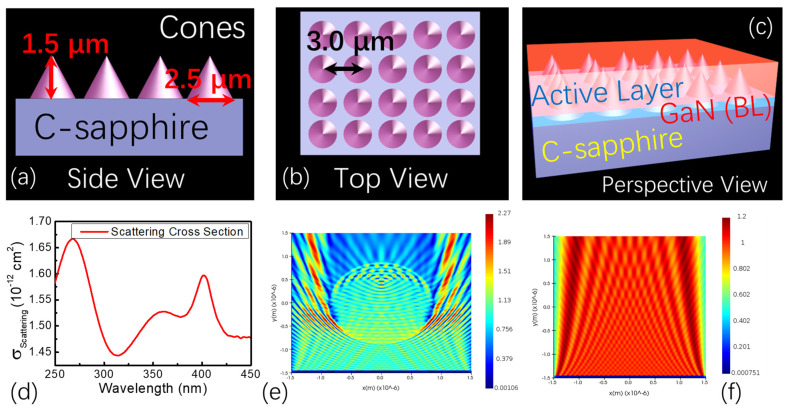
The schematic illustration of the undoped, Si-doped, and Mg-doped GaN thin films: (**a**) side view, (**b**) top view, (**c**) perspective view; (**d**) scattering cross-section of the thin films on hexagonal lattice arrangement patterned sapphire substrates calculated by using finite difference time domain method; electric field distribution in (**e**) the hexagonal lattice arrangement PSS and (**f**) FSS.

Before exploring the lasing action in the samples, the scattering-cross section of the hexagonal lattice arrangement patterned sapphire structure was calculated first. As seen in Figure 4a–c, the hexagonal lattice arrangement patterned sapphire structure was periodically aligned with the height of 1.5 μm and the distance of 2.5 μm. The bottom diameter of the cone was 2.0 μm. The multireflection, scattering, and coherent resonance of light in the PSS structure were analyzed by using the finite difference time domain method (FDTD, Ansys Lumerical 2020 R2 software, Ansys Inc., San Jose, CA, USA). Periodic c-sapphire cone arrays with a bottom radius of 2.5 µm, a height of 1.5 µm, and a distance of 3.0 µm were built, and a default refractive index of 1.792 nm was set at 370 nm [40]. The cones were surrounded uniformly by the GaN thin films with a refractive index of 2.661 at 370 nm [41]. The boundaries in the x-axis and y-axis were set as periodic conditions, and the boundary in the z-axis was selected as a perfect matched layer (PML). A plane wave incident to the arrays from the y-axis had the source range of 250 to 450 nm. The built-in scattering cross-section module was applied to calculate the scattering cross-section of the structure, as seen in Figure 4d; the most intense peak of the scattering cross-section appeared near 266 nm, and a broad scattering spectrum at 310 to 450 nm was also observed; this was a very desirable structure to perform lasing emission around 370 nm when it was pumped by a nanosecond laser around 266 nm at the same time. The electric field distribution around 370 nm in the periodic patterned structure and flat substrate was also plotted, as seen in Figure 4e,f; it was redistributed compared to the initial uniformly distributed electric field component of the incident light since light multireflection, scattering, and coherent resonance could occur within the periodic structures. The calculation and analysis implied that this structure was good for improving the performance and efficiency of the optically pumped photoluminescence and the lasing action in the samples, which was proved by experimental results in Figure S3 in SI. No lasing emission was observed; only broadband photoluminescence spectra were observed in the GaN on FSS even at very high pumping laser energy, as seen in Figure S4 in SI. It implied that the scattering and resonance of light within the PSS played a crucible role in the lasing emission of the GaN and the Mg-doped GaN thin films.

**Figure 5:**
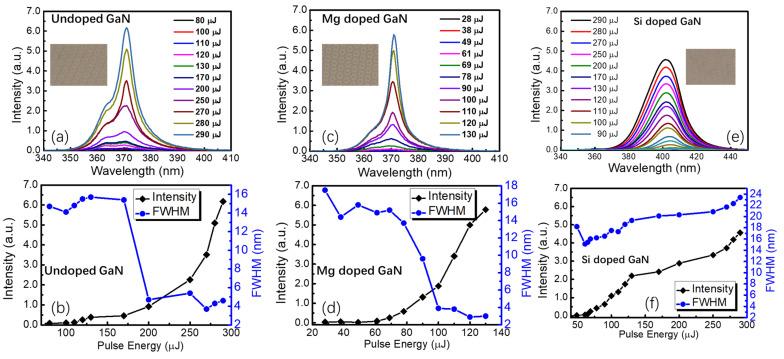
The fluorescence and lasing emission spectra of (**a**) undoped GaN, (**c**) Mg-doped GaN, and (**e**) Si-doped GaN thin films grown on the hexagonal lattice patterned periodic sapphire substrates (the insets are the molar pattern taken by a metallographic microscope); the lasing or fluorescence intensity and full width at half maximum (FHWM) varied along with the pumping pulse energy in (**b**) undoped GaN, (**d**) Mg-doped GaN, and (**f**) Si-doped GaN thin films grown on the hexagonal lattice patterned periodic sapphire substrates.

To investigate the lasing properties of the samples, a nanosecond laser at 266 nm was used as the excitation source. When the pulse laser was irradiated on the undoped GaN thin films, the broadband photoluminescence spectra with a full width at half maximum (FWHM) of approximately 16 nm were obtained below the threshold, as seen in Figure 5a. As the laser pulse energy exceeded 170 μJ, an asymmetric photoluminescence spectrum was observed and a sharp peak appeared at the top of the emission peak with a half-peak width of approximately 5 nm or narrower, indicating the formation of resonant radiative lasing emission. As shown in Figure 5b, the intensity of the lasing emission in undoped GaN thin films increased dramatically once the pulse laser energy exceeded the threshold. Compared with the undoped GaN thin films, the lasing emission in the Mg-doped GaN exhibited a lower threshold of 69 μJ and a narrower FWHM of approximately 3 nm (refer to Figure 5c,d). However, no lasing emission and spectrum narrowing phenomenon could be observed in the Si-doped GaN thin films, as drawn in Figure 5e,f; the values of the FWHM became even larger at a higher pulse energy, followed by the slowdown in peak intensity enhancement. The peak center of the photoluminescence showed a blue shift from 406.7 to 401.3 nm and implied the existence of the migration of ions in the active layer with significant thermal losses. As the increasing energy of 266 nm nanosecond pumping laser, no lasing emission but only broadband photoluminescence spectra were observed in the thin films on FSS even at very high pumping laser energy, as seen in Figure S5. It implied that the scattering and resonant of light within the PSS played a crucible role in the lasing emission of GaN and Mg doped GaN thin films.

## 4. Discussion

To discuss the physical picture behind the lasing action and fluorescence emission in the samples, the carrier concentrations, carrier types, and Hall coefficients of the samples were measured by using the Hall effect measurement system, as listed in Table 2. The undoped GaN thin film exhibited the intrinsic *n*-type carrier conductivity with the Hall coefficient of −8.08 × 10^4^ cm^3^/C and the carrier concentration of 7.73 × 10^13^/cm^3^. The Si-doped GaN thin film also exhibited the *n*-type carrier conductivity with the Hall coefficient of −0.103 cm^3^/C and the carrier concentration of 6.08 × 10^18^/cm^3^, while the Mg-doped GaN thin film showed the *p*-type carrier conductivity with the Hall coefficient of 9.41 cm^3^/C and the carrier concentration of 6.60 × 10^17^/cm^3^.

**Figure 6:**
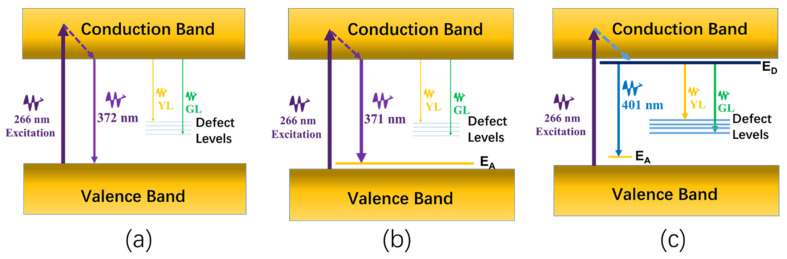
The energy transition diagram of the photoluminescence and lasing emission in (**a**) undoped GaN, (**b**) Mg-doped GaN, and (**c**) Si-doped GaN thin films.

The energy transition diagram describing the photoluminescence and lasing emission in *n*-type and *p*-type semiconductors was drawn in Figure 6. A typical intrinsic semiconductor band structure of the undoped GaN thin film was illustrated in Figure 6a; when the pulse laser was irradiated on the undoped GaN thin film, the electrons were excited into the conduction band and decayed to the lowest level of the conduction band rapidly through the nonradiative transition. In addition, the electrons recombined with holes on the valence band with a radiative light emission at 372 nm. At the same time, the weak YL and GL defect luminescence emission could be detected since a few of the Ga_N_ and N_Ga_ defects and the defect complex of V_Ga_-O_N_ existed in the thin film [32]. Some portion of the radiative photons around 372 nm could run out of the undoped GaN thin film immediately and be detected by the spectrometer, while the others were scattered and localized within the periodic cone structures. The quasi-three-level energy diagram and the positive resonant feedback of the periodic structure contributed to the resonant lasing emission upon exceeding the threshold. Similarly to that of the undoped GaN thin film, the lasing emission in the Mg-doped GaN thin films showed a lower threshold and a narrower lasing emission spectrum. As seen in Figure 6b, the lower defect densities of the Ga_N_ and N_Ga_ defects and the defect complex of V_Ga_-O_N_ further reduced the loss of the population of the excited electrons. Apart from the role of the resonant periodic cone structure, the activation energy level in the *p*-type Mg-doped GaN thin film formed the quasi-four-level transition structure, which also played an extremely important role in the formation of the lasing action. Due to a large number of defects in the Si-doped GaN thin film, as seen in Figure 6c, the population of electrons in the excited state could not be kept very high, which was not conducive to the formation of the laser. Although the donor energy (E_D_) near the conduction band and a quasi-four-level system were formed, no lasing emission was observed.

As described above, the photoluminescence and lasing action properties in the undoped and the ion-doped GaN could be affected greatly by the bandgap, the recombination emission way, the carrier types, and the defects in the wide-bandgap semiconductors, and the in-depth study of their roles in them showed a great guiding significance for integrated semiconductor optoelectronic devices and high-performance lasers.

## 5. Conclusions

In conclusion, resonant lasing emissions around 370 nm were achieved in the GaN and the Mg-doped GaN thin films on PSS. No lasing emission was observed; only broadband photoluminescence spectra were observed in the Si-doped GaN thin film on the PSS and the GaN thin film on the FSS because of the high dense defects in the thin films and lack of reflection and structures on the FSS. The morphology of the thin films proved that the PSS effectively reduced the density of the V-pits, which is beneficial to the application of GaN and Mg-doped GaN thin films in resonant lasers and optoelectronic devices. The scattering cross-section of the periodic structure showed a scattering peak around 375 nm, closing to the lasing emission center, which implied that the PSS enhanced the multireflection, scattering, and coherent resonance of the light and reduced the threshold of lasing emission in the GaN and Mg-doped GaN thin films. The defect densities of the samples brought a huge loss in the population of excited electrons, and the carrier concentrations and types of the samples varied the lasing formation system from a quasi-three-level system to a quasi-four-level system in *p*-type Mg-doped GaN thin film, in which there was a lower threshold and a narrower emission spectrum. This work analyzed the lasing properties of GaN, Mg-doped, and Si-doped GaN thin films on PSS, which are beneficial for developing optoelectronics semiconductor devices and lasers.

## Figures and Tables

**Table 1:** Growth conditions of the undoped, Si-doped, and Mg-doped GaN thin films.

Sample	Procedures	Growth Temperature	Gas Flow	Growth Times
Undoped GaN Si-doped GaN Mg-doped GaN	Buffer Layer	520 °C	TMGa: 8.3 μmol/min NH_3_: 0.5 sccm	15 min
Undoped GaN	Active Layer	850 °C	TMGa: 8.3 μmol/min NH_3_: 0.5 sccm	120 min
Si-doped GaN			TMGa: 8.3 μmol/min NH_3_: 0.5 sccm SiH_4_: 0.7 μmol/min	
Mg-doped GaN			TMGa: 8.3 μmol/min NH_3_: 0.5 sccm Cp_2_Mg: 0.4 μmol/min	
Undoped GaN Si-doped GaN Mg-doped GaN	Annealing	750 °C	N_2_: 60 sccm	5 min

**Table 2:** Hall coefficient, carrier types, and concentrations in undoped, Si-doped, and Mg-doped GaN thin films.

Sample	Magnet Strength (mT)	Hall Coefficient (cm^3^/C)	Carrier Concentration (cm^−3^)	Carrier Type
Undoped GaN	600	−8.08 × 10^4^	7.73 × 10^13^	*n*-type
Si-doped GaN	600	−0.103	6.08 × 10^18^	*n*-type
Mg-doped GaN	600	9.41	6.60 × 10^17^	*p*-type

## Data Availability

Not applicable.

## Supplementary Materials

The following supporting information can be downloaded at: https://www.mdpi.com/article/10.3390/nano12183238/s1, Figure S1. High magnification V-pits photos of Si doped GaN taken by scanning electron microscope. Figure S2. High magnification V-pits photos of a) Si doped and b) Mg doped GaN thin films taken by scanning electron microscope. Figure S3. The surface appearance of the GaN thin films on PSS and FSS. Figure S4. Photoluminescence spectra measured in GaN on PSS (red line) and FSS (black line). Figure S5. Photoluminescence of the GaN thin film on FSS versus pumping energy.
