# Supplementary material for: Resonant Lasing Emission in Undoped and Mg-Doped Gallium Nitride Thin Films on Interfacial Periodic Patterned Sapphire Substrates
Source: Nanomaterials (Basel). 2022 Sep 18;12(18):3238. doi: 10.3390/nano12183238 (PMC9505499; doi:10.3390/nano12183238)
Supplement: Supplementary file 1 [file nanomaterials-12-03238-s001.zip › nanomaterials-1900153-supplementary.pdf]

Supplementary Materials

Resonant Lasing Emission in Undoped and Mg-Doped Gallium Nitride Thin Films on Interfacial Periodic Patterned Sapphire Substrates

Long Xu [1], Yuehan Cao [1], Tianwei Song [1] and Caixia Xu [2,*]

[1] Chongqing Key Laboratory of Micro & Nano Structure Optoelectronics, School of Physical Science and Technology, Southwest University, Chongqing 400715, China
[2] School of Primary Education, Chongqing Normal University, Chongqing 400700, China
* Correspondence: noendness@126.com

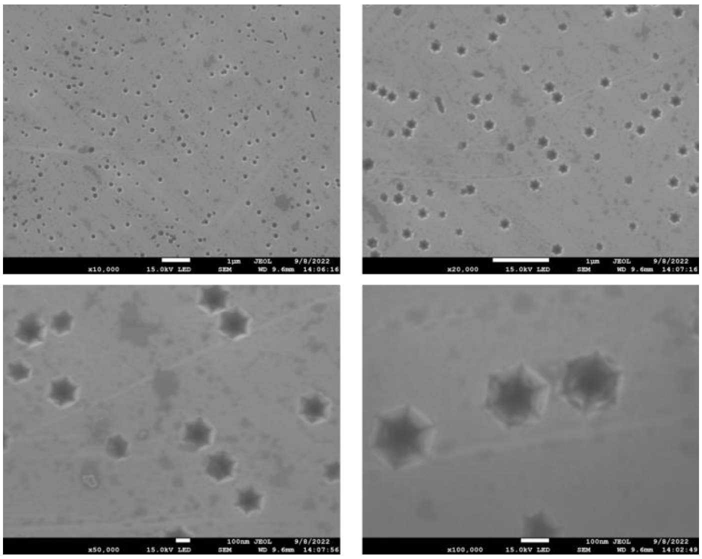

Figure S1. High magnification V-pits photos (a–d) of Si doped GaN taken by scanning electron microscope.

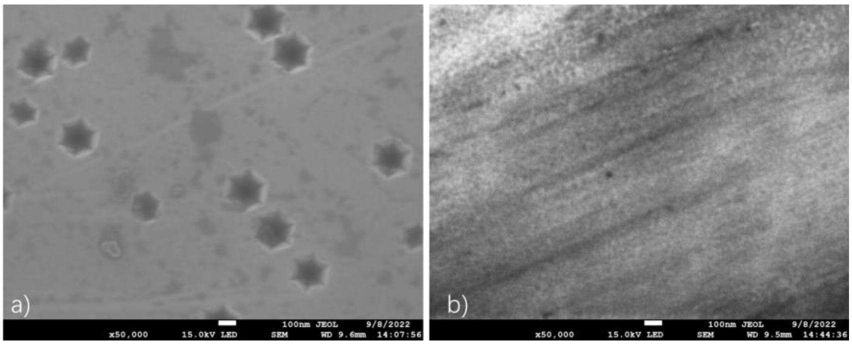

Figure S2. High magnification V-pits photos of (a) Si doped and (b) Mg doped GaN thin films taken by scanning electron microscope.

Very large but low dense V-pits were found in GaN thin film on FSS, as seen in Figure S3, which confirmed that the PSS improved the quality of the GaN thin films and reduce the dislocate mismatch between the substrate and the active layers.

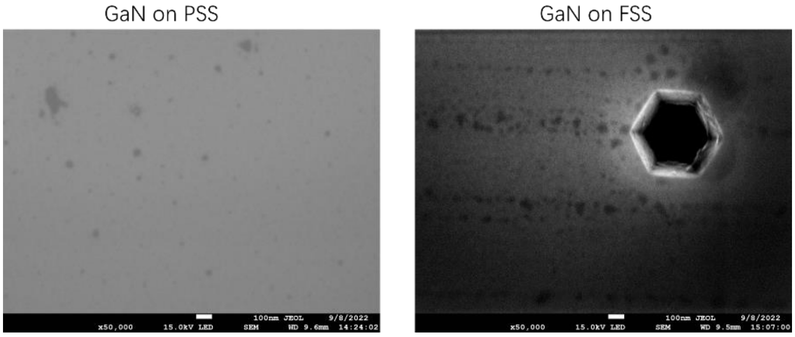

Figure S3. The surface appearance of the GaN thin films on PSS and FSS.

Over 100 times enhancement of the photoluminescence was obtained in GaN thin films on PSS than GaN thin films on flat sapphire substrate (FSS), as seen in Figure S4.

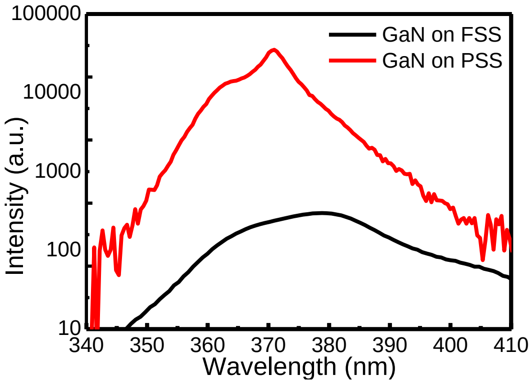

Figure S4. Photoluminescence spectra measured in GaN on PSS (red line) and FSS (black line).

As the increasing energy of 266 nm nanosecond pumping laser, no lasing emission but only broadband photoluminescence spectra were observed in the thin films on FSS even at very high pumping laser energy, as seen in Fig. S5.

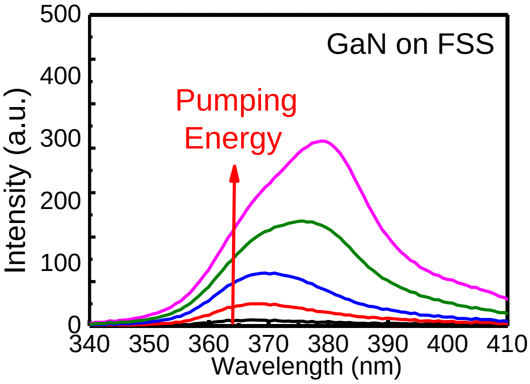

Figure S5. Photoluminescence of the GaN thin film on FSS versus pumping energy.
